# Employment of the Disabled

**Published:** 1920-04-24

**Authors:** 


### Employment of the Disabled.

A commendable record of what can be done for disabled ex-Service men is provided by Dr. Mead, who in a paper read before an international conference on the rehabilitation of the disabled, held under the auspices of the American Red Cross, gave some details regarding the Ford Motor Company. Dr. Mead, who is the Company's chief medical officer, said that nearly ten thousand men who are incapacitated have been accepted in the employ of the Company, and have been taught tasks which are within their capacity, so that they have become as efficient as normal men, and are, therefore, economically justified. The disabilities under which they suffer cover the whole range from loss or damage to hands, forearms, or damage to lower limbs, defective sight, loss of one or both eyes—there are at least four blind men amongst them. A number of men suffering from tuberculosis are put to work in an isolated building, and are at work that pays the Company 70,000 dollars per month. As these cripples qualify they are paid on the same basis as normal men. A further fact commented on by Dr. Mead is that light work is provided for convalescents in the Company's hospital, and the men thus supplement their disablement allowance and have a brighter time.

